# Case Report: GADA-positive type 1 diabetes with diabetic ketoacidosis in a young adult with obesity temporally associated with unsupervised tirzepatide use for weight reduction

**DOI:** 10.3389/fendo.2026.1995854

**Published:** 2026-10-22

**Authors:** Mengyun Lei, Fang Liu, Li Sun, Shuting Li, Juan Liu, Yonge Wang, Jun Tang

**Affiliations:** Department of Endocrinology, Zhongnan Hospital of Wuhan University, Wuhan, China

**Keywords:** case report, diabetic ketoacidosis, glutamic acid decarboxylase antibody, obesity, tirzepatide, type 1 diabetes

## Abstract

**Background:**

Tirzepatide, a dual glucose-dependent insulinotropic polypeptide/glucagon-like peptide-1 (GIP/GLP-1) receptor agonist, is widely used as an anti-obesity medication. Its safety in individuals with unrecognized type 1 diabetes (T1D) remains uncertain.

**Case description:**

We describe a 28-year-old man with obesity and no previously documented hyperglycemia who self-administered tirzepatide, lost 15 kg over about 3 months, and was later diagnosed with GADA-positive T1D presenting as diabetic ketoacidosis (DKA) in temporal association with drug use and discontinuation. At admission, fasting blood glucose (FBG) was 13.99 mmol/L, glycated hemoglobin (HbA1c) 10.8% (95 mmol/mol), serum β-hydroxybutyrate 4.44 mmol/L, urine ketones (+++), serum bicarbonate 16.9 mmol/L, glutamic acid decarboxylase antibody (GADA) >2000 IU/mL, and fasting C-peptide 0.798 ng/mL. Admission arterial blood gas was not obtained. Insulin therapy was initiated; HbA1c was 6.7% (50 mmol/mol) at 3 months.

**Conclusions:**

This single case cannot establish causality between tirzepatide exposure or discontinuation and autoimmune diabetes. It illustrates that adult-onset GADA-positive T1D with DKA may be recognized during or after unsupervised incretin-based weight reduction. Markedly elevated HbA1c indicated that hyperglycemia likely preceded the short symptomatic phase. The main clinical message is that autoimmune diabetes should be considered when unexplained hyperglycemia, ongoing weight loss, or ketoacidosis develops in this setting.

## Introduction

Tirzepatide, a novel dual agonist of glucose-dependent insulinotropic polypeptide (GIP) and glucagon-like peptide-1 (GLP-1) receptors, has been approved for type 2 diabetes (T2D) and obesity (1). It promotes insulin secretion, suppresses glucagon release, delays gastric emptying, and reduces appetite, resulting in substantial weight reduction (WR) (2).

Over recent decades, a growing number of individuals with type 1 diabetes (T1D) are overweight or obese. A large meta-analysis of 78 observational studies found that among adults with T1D, the prevalence of obesity increased from 3.3% in 1985–1990 to 23.7% in 2015–2020, with overweight prevalence reaching 31.4% (3). In the United States, data from the National Health Interview Survey (2016–2021) showed that 62% of adults with T1D had overweight or obesity (4).

This rising prevalence of excess weight in T1D is clinically important. Obesity in T1D exacerbates insulin resistance (IR), increases daily insulin requirements, and contributes to adverse cardiometabolic profiles, including a higher risk of cardiovascular disease and microvascular complications (5, 6). Consequently, adjunctive therapies that promote WR while maintaining glycemic control are increasingly being explored in T1D (7). Emerging evidence suggests that GLP-1 receptor agonists (GLP-1RAs), used alongside insulin, can improve glycemic control, reduce insulin needs, and support weight management in overweight or obese individuals with T1D (8).

However, the safety of these agents in individuals with unrecognized autoimmune diabetes remains incompletely understood. New-onset T1D has been reported after weight-loss use of semaglutide, including compounded formulations and online-prescribed semaglutide without pretreatment screening (9, 10). Reports describing tirzepatide in this setting are limited. A *post hoc* analysis of the SURPASS trials confirmed efficacy of tirzepatide in GADA-positive individuals with established T2D (11), but whether tirzepatide exposure or withdrawal is causally related to clinical recognition of autoimmune diabetes remains unknown.

We present a case of a young man with obesity and no previously documented hyperglycemia who developed severe hyperglycemia and diabetic ketoacidosis (DKA) in temporal association with unsupervised tirzepatide use and subsequent discontinuation, and was diagnosed with GADA-positive autoimmune T1D. The case is reported to highlight diagnostic vigilance rather than to claim a proven causal effect of tirzepatide.

## Case description

This case report was prepared according to the CARE guidelines (12). Written informed consent for publication was obtained from the patient.

A 28-year-old man presented with polydipsia, polyuria, and dry mouth for 5 days. He had no known diabetes. Two years earlier, fasting blood glucose(FBG) was 5.2 mmol/L on a routine health examination. About 6 months before admission he was obese (height 180 cm, weight 94 kg, body mass index [BMI] 28.9 kg/m²) with hypertriglyceridemia (3.95 mmol/L). Immediately before tirzepatide initiation, a random fingerstick glucose was 5.8 mmol/L. No recent laboratory FBG, glycated hemoglobin (HbA1c), islet autoantibodies, or C-peptide values were available before treatment; therefore, evolving dysglycemia cannot be excluded, and we describe the pretreatment status as no previously documented hyperglycemia rather than confirmed normoglycemia.

For WR, he self-purchased tirzepatide without medical supervision and injected it subcutaneously as follows: 2.5 mg once weekly for 4 weeks, then 5 mg once weekly for 4 weeks, then 7.5 mg once weekly for 4 weeks (approximately 12 weeks of weekly escalation), with 15 kg WR (94 kg to 79 kg; BMI 28.9 to 24.3 kg/m²). He then used 5 mg every 2–3 weeks for about 4 weeks as intermittent maintenance/tapering. He took the last tirzepatide dose at the end of this taper and then discontinued the drug completely after reaching his target weight. During tapering and after cessation, weight fell by a further 2 kg and appetite rebounded. Approximately 4 weeks after the last dose/complete cessation, osmotic symptoms began; after 5 days of symptoms he was admitted. His grandfather and several of his grandfather’s siblings had T2D; no autoimmune diseases were reported.

On admission, weight was 75 kg (BMI 23.15 kg/m²), vital signs were normal, and there was no Kussmaul breathing. The clinical timeline from tirzepatide initiation to diagnosis and follow-up is presented in **Figure 1**.

**Figure 1:**
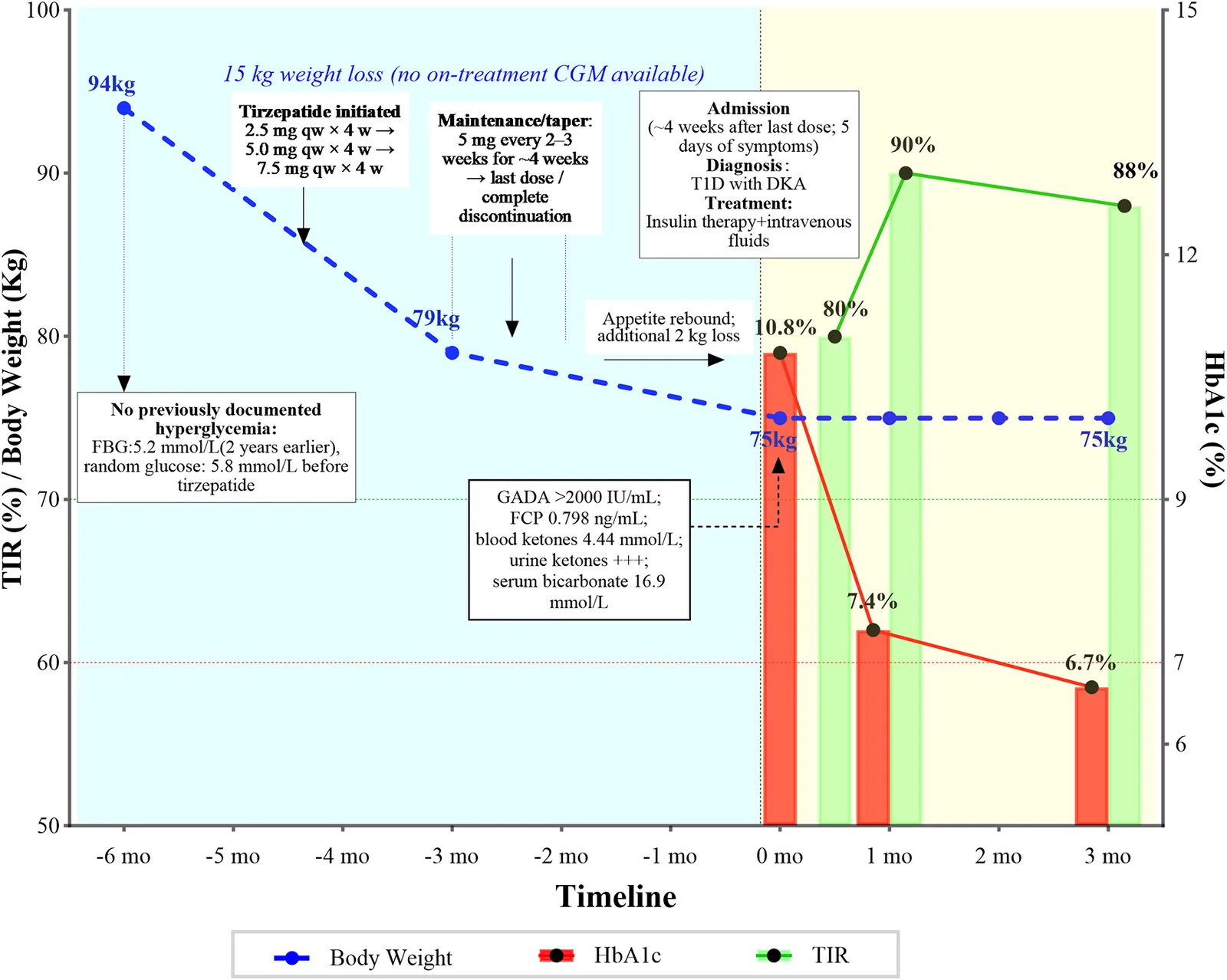
Clinical course from pretreatment assessment to 3-month follow-up. Body weight (kg), HbA1c (%), and CGM-derived time in range (TIR, %) are plotted against time relative to hospital admission (0 months). Key annotations: no previously documented hyperglycemia before tirzepatide (FBG 5.2 mmol/L two years earlier; random fingerstick glucose 5.8 mmol/L immediately before treatment); weekly dose escalation for ~12 weeks (2.5→5.0→7.5 mg), then ~4 weeks of intermittent 5 mg dosing, last dose/complete discontinuation, symptom onset ~4 weeks later, and admission after 5 days of symptoms with GADA-positive T1D and DKA (GADA >2000 IU/mL; fasting C-peptide 0.798 ng/mL; β-hydroxybutyrate 4.44 mmol/L; urine ketones +++; serum bicarbonate 16.9 mmol/L). Admission HbA1c was 10.8%, consistent with hyperglycemia preceding the symptomatic period and possibly preceding discontinuation. The figure shows temporal association only and does not establish causality between tirzepatide exposure or discontinuation and autoimmune diabetes. HbA1c, glycated hemoglobin; CGM, continuous glucose monitoring; TIR, time in range; FBG, fasting blood glucose; GADA, glutamic acid decarboxylase antibody; T1D, type 1 diabetes; DKA, diabetic ketoacidosis.

### Diagnostic assessment

Laboratory measurements at admission are summarized in **Table 1**.

**Table 1:** Laboratory test results at admission.

Variable	Value	Reference range	Comment
Glycemic control			
FBG (mmol/L)	13.99	3.9-6.1	High
2-h PG(mmol/L)	25.62	3.9-7.8	High
HbA1c	10.8% (95 mmol/mol)	4.0-6.5%	High
β-cell function			
FCP (ng/mL)	0.798	1.06-4.81	Low
2-h PCP (ng/mL)	1.076	2-5×fasting	Low
Autoantibodies			
GADA (IU/mL)	>2000	<10	High
ICA (U/mL)	129	<29.79	High
IAA (AU/mL)	16.6	<20	Normal
Ketosis and acid-base			
Serum β-hydroxybutyrate (mmol/L)	4.44	0-0.28	High
Urine ketones	+++	Negative	High
Serum Na^+^(mmol/L)	137.0	137-147	Normal
Serum Cl^-^(mmol/L)	100.4	99-110	Normal
Serum bicarbonate (mmol/L)	16.9	21-29	Low
Lipid profile			
TG (mmol/L)	3.95	<1.7	High
TC (mmol/L)	4.08	<5.18	Normal
LDL-c (mmol/L)	1.65	<3.40	Normal
HDL-c (mmol/L)	0.51	>1.04	Low
Renal function			
Uric acid (μmol/L)	688.7	208-428	High
eGFR (mL/min/1.73 m²)	132.24	>90	Normal
UACR (mg/mmol)	1.24	0-3	Normal
Thyroid function			
FT3 (pmol/L)	1.676	1.92-4.44	Low
FT4 (pmol/L)	15.49	10.51-22.7	Normal
TSH (mIU/L)	1.912	0.25-4.3	Normal
TgAb	Negative	—	Normal
TPOAb	Negative	—	Normal

FBG, fasting blood glucose; 2-h PG, 2-hour postprandial glucose; FCP, fasting C-peptide; 2h-PCP, 2-hour postprandial C-peptide; GADA, glutamic acid decarboxylase antibody; ICA, islet cell antibody; IAA, insulin autoantibody; TG, triglycerides; TC, total cholesterol; LDL-c, low-density lipoprotein cholesterol; HDL-c, high-density lipoprotein cholesterol; eGFR, estimated glomerular filtration rate; UACR, urine albumin-to-creatinine ratio; FT3, free triiodothyronine; FT4, free thyroxine; TSH, thyroid-stimulating hormone; TgAb, thyroglobulin antibody; TPOAb, thyroid peroxidase antibody.

Key findings included: (1) hyperglycemia—FBG 13.99 mmol/L, 2-hour glucose 25.62 mmol/L after a standardized steamed-bun meal test (100 g steamed bun, approximately 50–55 g available carbohydrate, used locally as a standardized mixed-carbohydrate stimulus), HbA1c 10.8% (95 mmol/mol); (2) impaired β-cell function—fasting C-peptide (FCP) 0.798 ng/mL with concurrent FBG 13.99 mmol/L, and 2-hour postprandial C-peptide 1.076 ng/mL, indicating a blunted C-peptide response relative to marked hyperglycemia; (3) positive autoantibodies—GADA >2000 IU/mL and islet cell antibody (ICA) 129 U/mL; and (4) DKA at admission—serum β-hydroxybutyrate 4.44 mmol/L, urine ketones (+++), serum sodium 137.0 mmol/L, chloride 100.4 mmol/L, and serum bicarbonate 16.9 mmol/L (anion gap 19.7 mmol/L). Arterial blood gas was not obtained at admission. On hospital day 3, after fluids and insulin and after ketones had normalized, arterial pH was 7.45, with actual bicarbonate 28.5 mmol/L, reflecting recovery.

Despite only 5 days of osmotic symptoms, the HbA1c of 10.8% indicated that hyperglycemia had likely been present for weeks to months and may have preceded tirzepatide discontinuation. According to the 2024 hyperglycemic crises consensus, glucose ≥11.1 mmol/L, ketosis, and metabolic acidosis fulfilled biochemical criteria for DKA (13). Pretreatment GADA and C-peptide were not measured—an important limitation that prevents determining whether autoimmunity and β-cell failure were already present before tirzepatide exposure.

### Therapeutic intervention

The patient received intravenous fluids, continuous subcutaneous insulin infusion (CSII), continuous glucose monitoring (CGM), and 1 week of structured T1D education. Ketones normalized by day 3, at which time arterial blood gas showed pH 7.45 and actual bicarbonate 28.5 mmol/L, consistent with resolution of ketoacidosis; CSII was then transitioned to multiple daily insulin injections (MDI). He was discharged on MDI with resolved symptoms and stable glucose control.

### Follow-up and outcomes

At discharge, CGM-derived time in range (TIR, 3.9–10.0 mmol/L) reached 80%. At the 1-month follow-up, HbA1c was 7.4% (57 mmol/mol) with a TIR of 90%. At 3-month follow-up, HbA1c improved to 6.7% (50 mmol/mol) with a TIR of 88% and he was on a stable insulin regimen (**Figure 2**).

**Figure 2:**
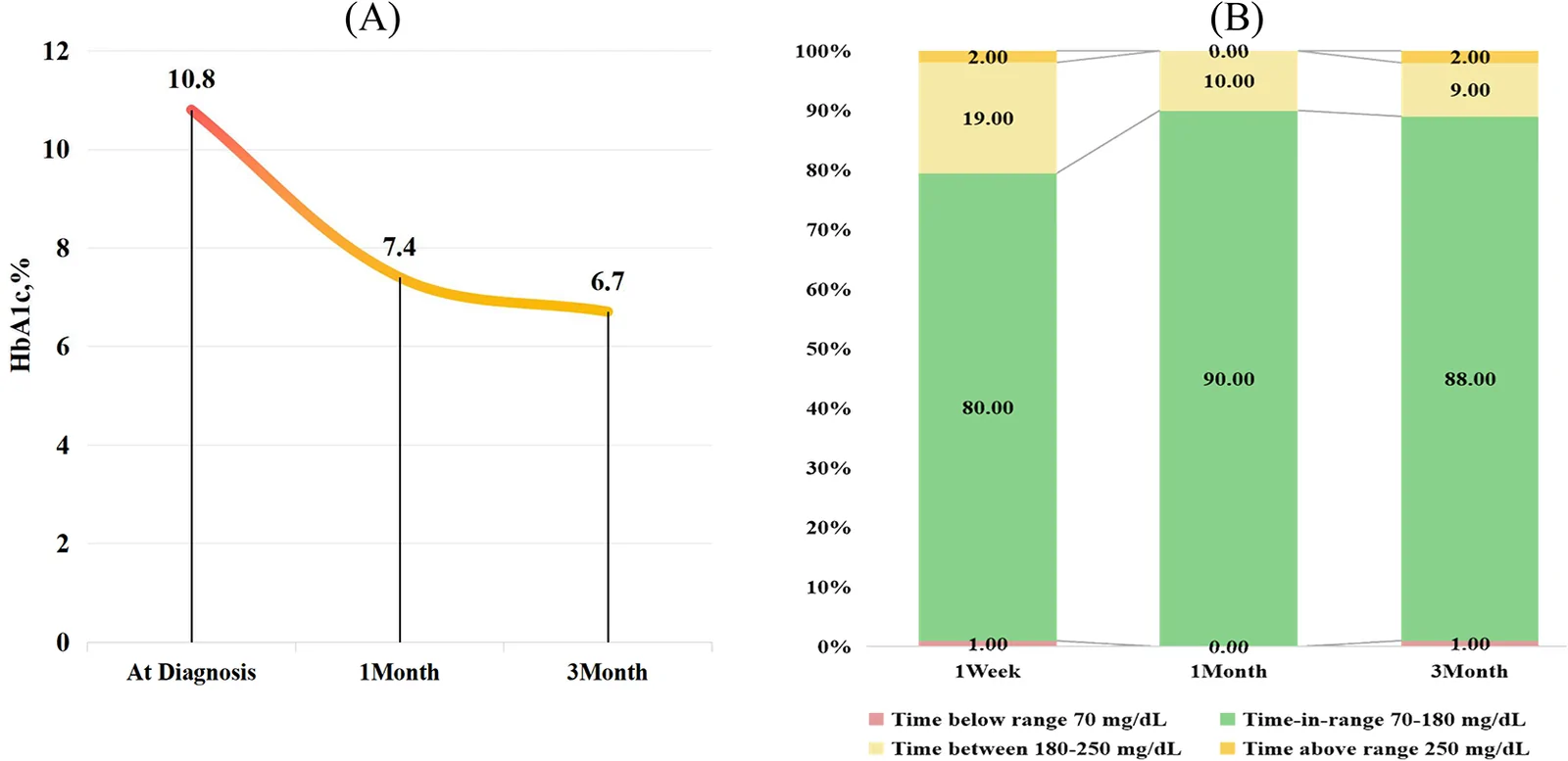
Glycemic control after insulin therapy. **(A)** HbA1c fell from 10.8% (95 mmol/mol) at diagnosis to 7.4% (57 mmol/mol) at 1 month and 6.7% (50 mmol/mol) at 3 months. **(B)** CGM profiles at 1 week, 1 month, and 3 months: TBR <3.9 mmol/L (1%, 0%, and 1%); TIR 3.9–10.0 mmol/L (80%, 90%, and 88%); and TAR, shown as 10.0-13.9 mmol/L plus >13.9 mmol/L, totaling 19%, 10%, and 11%, respectively. CGM, continuous glucose monitoring; HbA1c, glycated hemoglobin; TAR, time above range; TBR, time below range; TIR, time in range.

## Discussion

A 28-year-old man with no previously documented hyperglycemia was diagnosed with DKA and GADA-positive autoimmune T1D in temporal association with unsupervised tirzepatide use and discontinuation. At presentation, FBG was 13.99 mmol/L, HbA1c 10.8%, ketones were markedly elevated, admission serum bicarbonate was 16.9 mmol/L, GADA was strongly positive (>2000 IU/mL), and fasting C-peptide was low (0.798 ng/mL) despite concurrent hyperglycemia. These findings support autoimmune T1D rather than T2D, despite obesity and a family history of T2D.

The phenotype of new-onset T1D has changed. Obesity and a family history of T2D no longer exclude autoimmune diabetes (14, 15). In this patient, DKA and strongly positive GADA were key to the diagnosis.

Incretin-based therapies have well-established glycemic and weight benefits in T2D and obesity, with emerging evidence as insulin adjuncts in T1D (8, 16–20). A *post hoc* analysis of SURPASS also showed benefit in GADA-positive individuals with established T2D (11). To contextualize our findings, **Table 2** summarizes previously reported cases for comparison; the list is not exhaustive and is not based on a systematic review. Importantly, tirzepatide-associated starvation or hypoglycemic ketoacidosis without autoimmunity (21, 22) should be distinguished from new-onset autoimmune T1D (9, 10). Most previously reported ketoacidosis or ketosis during tirzepatide use occurred in non-diabetic weight-loss settings, often with gastrointestinal symptoms, reduced oral intake, or hypoglycemia, and without autoimmune diabetes (21, 22). Euglycemic diabetic ketoacidosis has been described with another GLP-1RA in established T2D (23). New-onset autoimmune T1D has also been reported after compounded semaglutide (9) and after online-prescribed semaglutide without pretreatment screening (10). The latter case resembles ours in unsupervised obesity treatment and missing pretreatment screening, but our patient used tirzepatide, presented with DKA, and was diagnosed after discontinuation rather than during ongoing therapy.

**Table 2:** Previously reported cases of ketosis/ketoacidosis or new-onset autoimmune T1D after incretin-based therapies (for comparison; not exhaustive).

Authors, year	Drug	Patient characteristics	Precipitating factors	Clinical presentation	Diagnosis	Autoantibodies	Outcome
Bitar Z et al., 2024 (21)	Tirzepatide	29-year-old woman, non-diabetic, BMI 32 kg/m²	Dose increase to 5 mg in week 5	Hypoglycemic ketoacidosis with vomiting	Starvation ketosis	NR	Recovered
Bitar Z et al., 2024 (21)	Tirzepatide	34-year-old woman, non-diabetic, BMI 31.3 kg/m²	Dose increase to 5 mg	Hypoglycemic ketoacidosis (glucose 2.8 mmol/L)	Starvation ketosis	NR	Recovered
Bitar Z et al., 2024 (21)	Tirzepatide	17-year-old girl, non-diabetic, BMI 30.4 kg/m²	Week 5 of treatment	Hypoglycemic ketoacidosis with GI symptoms	Starvation ketosis	NR	Recovered
Bitar Z et al., 2024 (21)	Tirzepatide	26-year-old woman, non-diabetic, BMI 30.8 kg/m²	After 2 weeks of 5 mg dose	Hypoglycemic ketoacidosis (glucose 3.1 mmol/L)	Starvation ketosis	NR	Recovered
Iqbal PMR et al., 2024 (22)	Tirzepatide	21-year-old woman, non-diabetic, BMI 28.2 kg/m²	5 mg weekly for 3 weeks	Ketoacidosis (pH 7.22; ketones 5.2 mmol/L; glucose 4.9 mmol/L)	Starvation ketosis	NR	Recovered
Alduraibi RK et al., 2023 (23)	Dulaglutide	45-year-old man with T2D	Low-carb diet; GI symptoms; first dulaglutide dose; concurrent SGLT2 inhibitor	Euglycemic DKA (pH 6.95; bicarbonate 4 mmol/L)	EDKA in T2D	GADA negative	Recovered
Bueso A et al., 2025 (9)	Semaglutide (compounded)	62-year-old woman (GBS history)	Weight-loss use	DKA	New-onset T1D	GAD-65+, ICA+	New-onset T1D
Bueso A et al., 2025 (9)	Semaglutide (compounded)	35-year-old woman (GDM history)	Weight-loss use	DKA	New-onset T1D	GAD-65+	New-onset T1D
Hasse L et al., 2026 (10)	Semaglutide (Wegovy)	49-year-old woman, non-diabetic, BMI 29.4 kg/m²	Online prescription without prior screening	Hyperglycemia from month 4; no ketoacidosis at presentation	New-onset T1D	GAD-65+, low C-peptide	New-onset T1D

BMI, body mass index; DKA, diabetic ketoacidosis; EDKA, euglycemic diabetic ketoacidosis; GADA, glutamic acid decarboxylase antibody; GBS, Guillain-Barré syndrome; GDM, gestational diabetes mellitus; GI, gastrointestinal; GLP-1RA, glucagon-like peptide-1 receptor agonist; ICA, islet cell antibody; NR, not reported; T1D, type 1 diabetes; T2D, type 2 diabetes. Bueso et al. (9) is a meeting abstract. Starvation/hypoglycemic ketoacidosis without autoimmunity (Bitar Z et al.; Iqbal PMR et al.) is etiologically distinct from new-onset autoimmune T1D (Bueso A et al.; Hasse L et al.).

We cannot establish causality from a single case. One possible relationship is that substantial WR and improved insulin sensitivity during tirzepatide use temporarily masked progressive β-cell failure, with clinical recognition around the time of drug discontinuation; alternatively, autoimmune T1D may have progressed independently, and the timing with discontinuation may be coincidental. These explanations remain hypotheses. Critically, the absence of pretreatment GADA and C-peptide measurements is a major limitation: we cannot determine whether autoimmunity and limited β-cell reserve were already present before tirzepatide exposure, nor can we demonstrate that discontinuation precipitated decompensation. The HbA1c of 10.8% further indicates that hyperglycemia likely antedated the 5-day symptomatic phase and may have begun before complete drug withdrawal. Strongly positive GADA at diagnosis supports autoimmune T1D, but the onset of autoimmunity is unknown. In the semaglutide report by Hasse et al., symptoms developed during ongoing therapy and were considered possibly coincidental (10).

At age 28 years, with strongly positive GADA, DKA, low but detectable C-peptide, and immediate need for insulin, the presentation is best described as adult-onset autoimmune T1D rather than latent autoimmune diabetes in adults, which typically has a more insidious course and delayed insulin requirement (14, 15).

This case report has several limitations: (1) single-case design precludes causal inference; (2) most importantly, pretreatment GADA and C-peptide were not measured, so pre-existing autoimmunity cannot be confirmed or excluded; (3) no recent pretreatment HbA1c was available, and the admission HbA1c precludes precise timing of hyperglycemia relative to drug withdrawal; (4) admission arterial blood gas was not obtained, so arterial pH at presentation was unavailable (DKA was diagnosed from admission serum bicarbonate 16.9 mmol/L); and (5) HLA typing was not performed.

Take-away lessons: Incretin-based weight-loss drugs remain important therapies under clinical supervision, and this case does not justify avoiding tirzepatide in appropriate candidates. The stronger message is that autoimmune diabetes should be considered when unexplained hyperglycemia, continued weight loss, or ketoacidosis develops during or after incretin-based weight management, even in young adults with obesity and no previously documented hyperglycemia. Glucose monitoring and prompt evaluation are reasonable when such symptoms occur; however, a single case cannot support routine post-discontinuation monitoring or routine pretreatment autoantibody/C-peptide testing for all users. If autoimmune diabetes is confirmed, insulin should be started promptly and weight-loss drugs monotherapy avoided.

### Patient perspective

I am 28 years old. I learned about tirzepatide online, which was being widely promoted as a popular weight-loss ‘miracle drug’, and bought it online and used it on my own, without seeing a doctor first. My blood glucose had always been normal on previous check-ups, so diabetes never crossed my mind.

The injections worked quickly. I lost 15 kg in about 3 months and was happy with the result. After I reached my target weight, I slowly stopped the medicine.

About 1 month later, I became extremely thirsty, drunk much and needed to urinate constantly. I kept losing weight even though I was no longer taking tirzepatide. I thought it might be a normal reaction to stopping the drug. When the symptoms worsened, I came to the hospital and learned I had T1D with DKA. I was surprised—I had just lost weight successfully and did not think diabetes could present this way.

I now wish I had seen a doctor before starting tirzepatide and had monitored my blood glucose during treatment and after stopping it. Insulin therapy and the education I received in hospital have helped me adjust. Three months later, my HbA1c is 6.7% and CGM has made day-to-day management easier.

## Data Availability

The original contributions presented in the study are included in the article/supplementary material. Further inquiries can be directed to the corresponding authors.
